# Supplementary material for: The Mycobacterium bovis BCG GroEL1 Contributes to Isoniazid Tolerance in a Dormant-Like State Model
Source: Microorganisms. 2023 Jan 21;11(2):286. doi: 10.3390/microorganisms11020286 (PMC9966693; doi:10.3390/microorganisms11020286)
Supplement: Supplementary file 1 [file microorganisms-11-00286-s001.zip › microorganisms-2163416-supplementary.pdf]

**Supplementary materials**

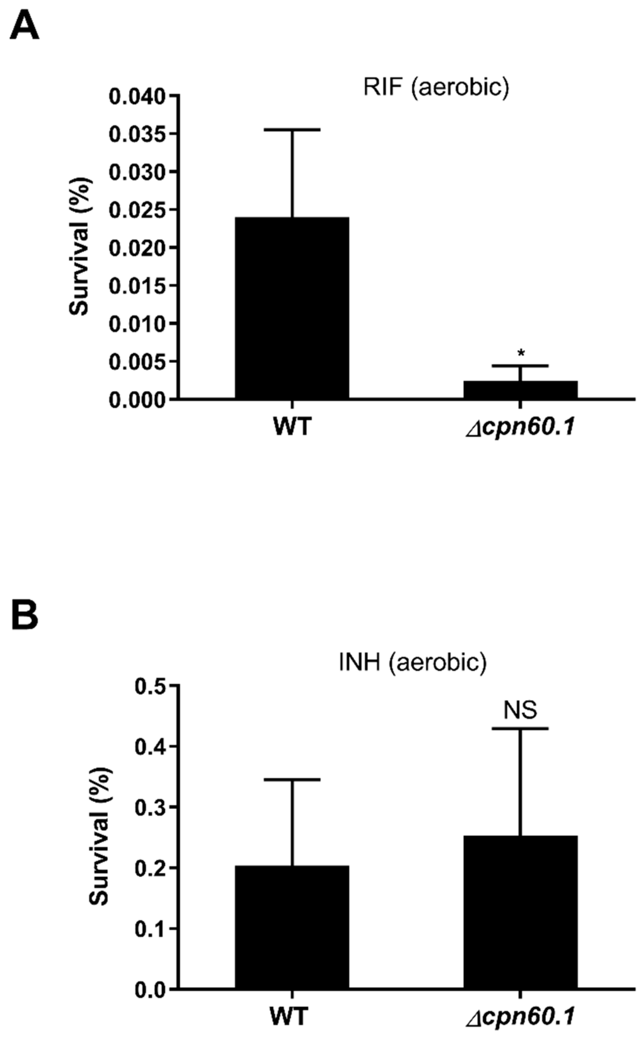


**Figure S1.** Loss of GroEL1 increased RIF susceptibility, but has no impact on INH in aerobic cultures. *M. bovis* BCG cultures grown aerobically were treated with 0.1 µg/mL RIF (A) or with 0.4 µg/mL INH (B) for 5 days. Viability was determined by plating proper dilutions on 7H11 agar plates and compared with that prior to drug treatment. Mean values and standard deviations from three or four experiments are shown. *, $p<0.05$; NS, statistically no significant difference.

A

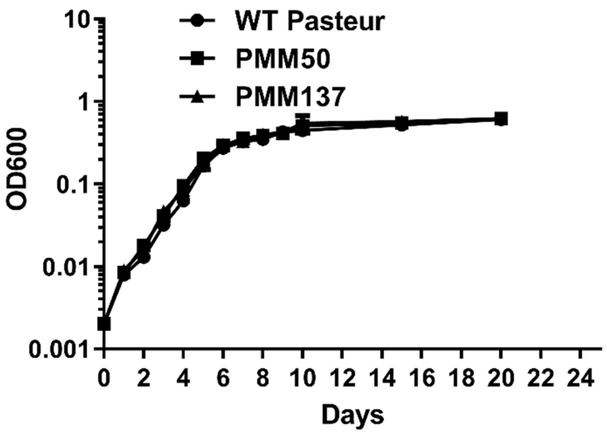


B

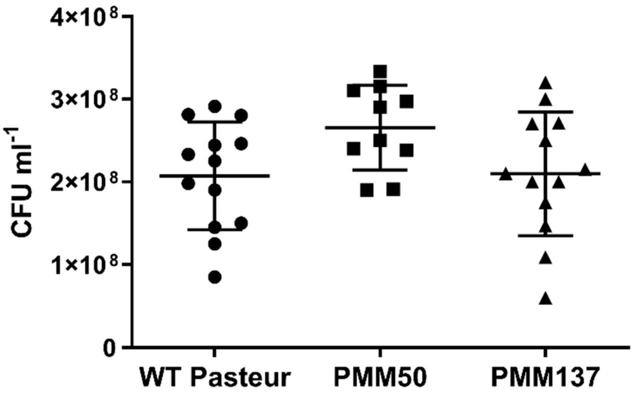


**Figure S2.** The loss of PDIM in the *M. bovis* BCG cell wall is not interfering with the establishment of growth arrest in the Wayne dormancy model. Growth curves of WT, PMM50 and PMM137 *M. bovis* BCG strains in the Wayne dormancy model (A). Viability of WT, PMM50 and PMM137 strains were determined at Wayne day 20 (B). Mean values from three independent experiments are shown.
